# The benefits of computer-assisted total knee arthroplasty on coronal alignment with marked femoral bowing in Asian patients

**DOI:** 10.1186/s13018-014-0122-3

**Published:** 2014-12-03

**Authors:** Chien-Yin Lee, Shih-Jie Lin, Liang-Tseng Kuo, Kuo-Ti Peng, Kuo-Chin Huang, Tsan-Wen Huang, Mel S Lee, Robert Wen-Wei Hsu, Wun-Jer Shen

**Affiliations:** Department of Orthopaedic Surgery, Chang Gung Memorial Hospital, 6, West Section, Chia-Pu Road, Pu-Tz City, Chia-Yi Hsien 613 Taiwan; Chang Gung University, 259 Wen-Hwa 1st Road, Kwei-Shan, Tao-Yuan 333 Taiwan; Po-Cheng Orthopaedic Institute, 100 Bo-ai 2nd Road Zuoying District, Kaohsiung, Taiwan

**Keywords:** Bowing deformity of femur, Arthroplasty, Knee, Replacement, Surgery, Computer-assisted

## Abstract

**Background:**

Mechanical alignment guides are designed to compensate for variations in the valgus alignment angle; however, these guides may not be adequate when a patient has coronal alignment with marked bowing deformity. Previous study demonstrates better radiographic results, but the clinical benefits are a matter of speculation. The aim of this study was to investigate whether radiographic benefits of computer-assisted surgery total knee arthroplasty (CAS-TKA) would translate to clinical outcomes.

**Methods:**

Patients with osteoarthritis and coronal alignment with marked bowing deformity who underwent total knee arthroplasty (TKA) at our institution between January 2005 and June 2012 were entered into this retrospective study. Patients were divided into three groups: patients with coronal alignment with marked bowing deformity treated with CAS-TKA; with coronal alignment with marked bowing deformity treated with conventional TKA; and without marked coronal bowing deformity treated with conventional TKA. The computer-assisted navigation and the conventional technique were then compared by radiographic parameters. The International Knee Society (IKS) scores and patellar score were obtained for all patients preoperatively and at the last follow-up visit.

**Results:**

One hundred and thirty-seven patients (198 knees) met the inclusion criteria. For patients with osteoarthritic knees with marked femoral bowing deformity, the reconstructed mechanical axis (MA) was significantly closer to normal in the CAS-TKA group (*P* = 0.002) than in the conventional group. Significant differences in the reconstructed MA after conventional TKA were noted between patients without bowing and those with bowing (*P* = 0.003). Using the patellar score and IKS score, at a mean follow-up of 52.2 months, the differences did not achieve statistical significance among the three groups.

**Conclusions:**

CAS-TKA was an effective alternative for obtaining proper alignment in patients with coronal alignment with marked bowing deformity. However, there was no statistically significant difference in clinical function between patients treated with CAS-TKA and conventional TKA. Long-term follow-up will be needed to determine if the improvement in radiographic results translates to better clinical outcomes.

## Introduction

Patient factors, prosthetic design, and surgical technique all affect the survivorship of total knee arthroplasty (TKA) [1–6]. Studies using finite element models as well as long-term survival studies confirm that the longevity of the implants and optimal long-term outcomes depend on the accuracy of bone cuts (within 3° from the ideal position) and proper restoration of the mechanical axis of the leg [7–12].

Current mechanical alignment guides for TKA have many limitations. Several authors have reported malaligned components in more than 25% of patients they used an intramedullary alignment system [13–15]. Deformities of the femur, such as the presence of diaphyseal deformity, distortion of the bony canal, and variations in femoral anatomy, are likely to further decrease the accuracy [13–17]. The prevalence of coronal alignment with marked bowing deformity in patients with end-stage osteoarthritis of the knee is particularly relevant in Asian populations because this deformity reportedly affects as many as 62% of Asians [18–20]. Such deformities will alter the desired angle between the mechanical and anatomical axis of the lower extremity and thereby jeopardize positioning of the femoral component and postoperative mechanical axis of the limb [18–22].

Computer-assisted surgery total knee arthroplasty (CAS-TKA) has been shown to improve component alignments and limb axis correction and to lessen gap asymmetry in patients with arthritic knees complicated by extra-articular deformities [23–25]. However, few studies have addressed the benefits of CAS-TKA in patients with coronal alignment with marked bowing deformity [18–21]. In 2011, we published a radiographic study to investigate the impact of marked femoral bowing on the placement of components and postoperative mechanical axis in TKA [6]. A total of 306 knees were compared and demonstrated that marked femoral bowing resulted in inaccuracy of the position of femoral component and limb alignment when a conventional technique was used. However, several limitations in the previous radiological study must be acknowledged. First, patients underwent primary TKA by three surgeons using different prostheses. Secondly, we were unable to assess any correlation between alignment and functional outcome. In order to further clarify the clinical influence of marked coronal femoral bowing and the role of CAS-TKA, we now report the comparisons of radiographic and clinical outcomes. All patients received the same total knee prosthesis (cruciate-retaining design press-fit condylar sigma fixed-bearing components [DePuy PFC Knee Systems, DePuy Orthopaedics, Warsaw, IN, USA]). All procedures were performed by the senior surgeon (Hsu RWW) who has extensive experience in the use of both conventional mechanical guides and computer-assisted navigation. The strict inclusion criteria for this investigation were designed to limit the variables under study. The purpose was to assess whether the CAS-TKA is more useful than the conventional TKA for patients with coronal alignment with marked bowing deformity. We hypothesize that CAS-TKA provides better clinical outcomes when marked coronal femoral bowing is presented.

## Methods

This retrospective study was approved by the Ethics Committee and Institutional Review Board of the Chang Gung Memorial Hospital (99-2025B). We searched our arthroplasty database and performed a manual review of patient records to identify all patients who received a primary TKA by a single experienced orthopedic surgeon (R.W.-W.H.) at our institution between January 2005 and June 2012. Patients were excluded for the following reasons: (a) having a minimum follow-up of less than 24 months; (b) having extra-articular deformity of the femur or tibia due to previous trauma or surgery, and (c) having incomplete medical records, radiographic analyses, or clinical functional assessments. Clinical data, including age, gender, diagnosis, length of hospital stay, operative procedures performed, tourniquet time, total amount of blood loss, complications associated with surgical technique, and radiographic as well as clinical functional assessments before and after surgery were collected.

**Figure 1:**
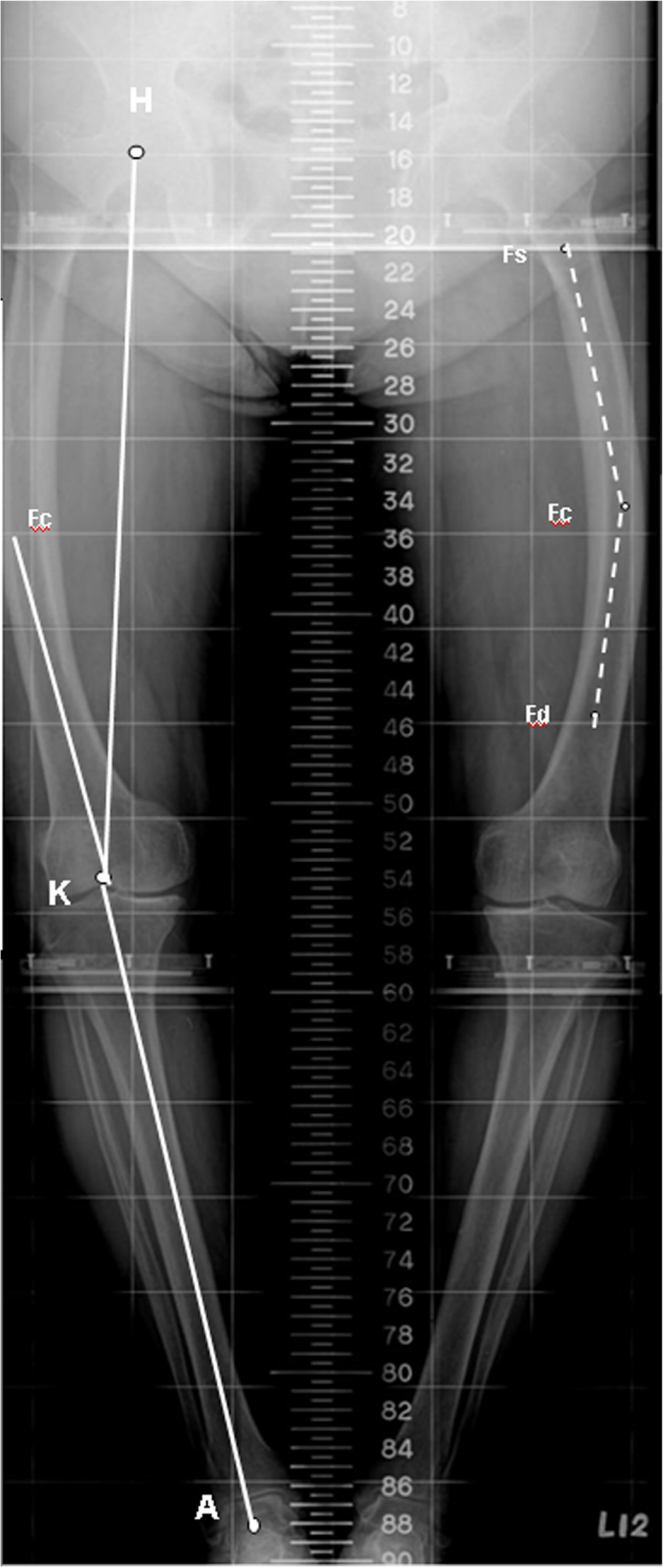
**This schematic diagram illustrates the key radiographic landmarks used to define the axial alignment parameters.** *H*, femoral head center; *K*, knee joint center; *A*, ankle joint center; *Fs*, midpoint of cortical width at lesser trochanter; *Fd*, a point bisecting the shaft 10 cm proximal to the knee joint. A point bisecting the shaft midway between *Fs* and *Fd* was designated as *Fc*; *H-K-A*, mechanical axis of the lower extremity; *HK-FcK*, the valgus correction angle of the distal femur (measured using the method described by Yau et al. [18]); *FsFc-FcFd*, the coronal femoral bowing angle.

All patients had preoperative and postoperative anteroposterior and lateral radiographs of the knees, along with full-length weight-bearing roentgenograms of the lower extremity (the method for this is described in detail in a previous publication [26] by the senior author). The magnitude of coronal femoral bowing was measured using the method of Mullaji et al. [19]. Coronal femoral bowing magnitude greater than 5° was identified as marked coronal femoral bowing deformity. Using Mullaji’s criteria, femoral bowing greater than 5° was considered substantial [20] (Figure 1).

**Figure 2:**
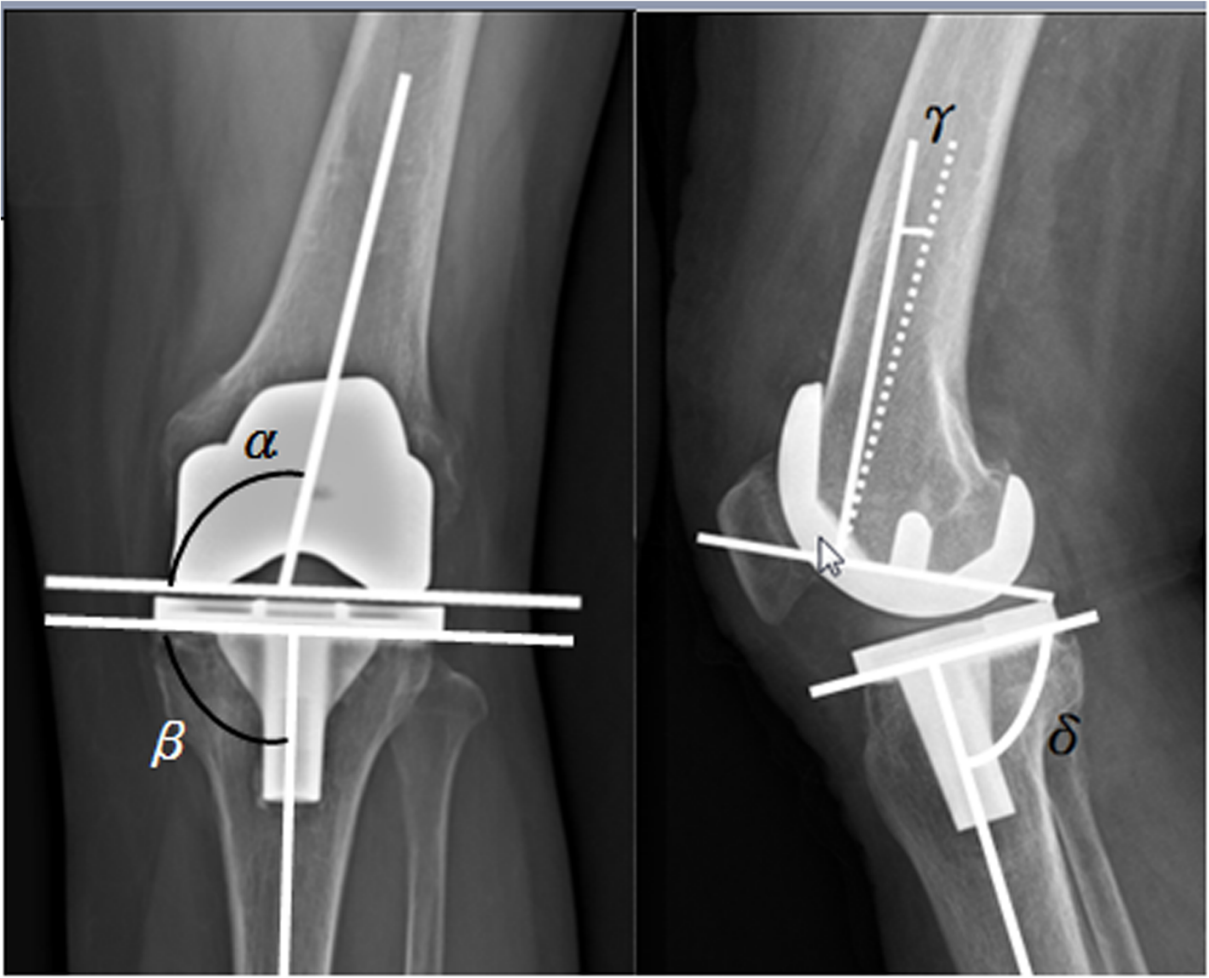
**Radiograph showing the measurement of four component alignment angles.** *α* = femoral valgus angle (FV), *β* = tibial valgus angle (TV), *γ* = femoral flexion angle (FF), and *δ* = tibial flexion angle (TF). The Knee Society total knee arthroplasty roentgenographic evaluation system originally appeared in [27].

Radiographic parameters, including the preoperative mechanical axis, postoperative mechanical axis, valgus correction angle of the distal femur, femoral bowing angle, and four component alignment angles, specifically the femoral valgus angle (FV), tibial valgus angle (TV), femoral flexion angle (FF), and tibial flexion angle (TF) were analyzed [27] (Figure 2). The goal was to reconstruct the coronal mechanical axis to within 3° of the ideal position. All digital radiographs were reviewed and measured by one independent observer (Yu-Shuan Lin) who was blinded to the surgical techniques, allocation of three groups, and patient’s demographic data. The intraobserver reliability was assessed according to the method described by Konigsberg et al. [28]. The intraobserver reliability was rated as good to very good.

Clinical outcomes were assessed using International Knee Society (IKS) scores [29] and the patellar score [30], which were checked preoperatively and at the last follow-up. Active maximum range of motion (ROM) of the knee was measured with a goniometer. All clinical outcomes were collected and analyzed by two independent surgeons who were blinded to the surgical technique.

All TKAs were performed using an anterior midline longitudinal skin incision and a medial parapatellar arthrotomy. The conventional TKA was implanted with use of conventional mechanical guides, with an intramedullary alignment guidance system for femoral preparation and an extramedullary guide for tibial preparation. The angle of the intramedullary aligned distal femoral cutting block was adjusted according to the valgus correction angle of the distal femur, which was measured by full-length weight-bearing roentgenograms of the lower extremity [18]. The CAS-TKA was implanted with the use of a computed tomography (CT)-free navigation system (BrainLAB AG, Munich, Germany).

Patients were divided into three groups. Group A included patients with coronal alignment with marked bowing deformity (greater than 5°, measured by Mullaji’s method and defined by Mullaji’s criteria) who underwent CAS-TKA. Patients with coronal alignment with marked bowing deformity who underwent conventional TKA were assigned to group B. Group C included patients without marked coronal bowing deformity (less than 5° measured by Mullaji’s method and defined by Mullaji’s criteria) who had undergone conventional TKA. To determine adequate sample size, an *a priori* power analysis using the hypothesis test with a power of 80% and a significance of 0.05 was done. It was calculated that 51 knees were required per group to detect a difference of 5 points in the Knee Society score (estimated SD of >8). The cutoff value was selected because a difference of 5 points has been suggested as the minimal clinically important difference for the Knee Society score [31].

All data were entered in an Excel spreadsheet (Microsoft, Redmond, WA, USA), rechecked for missing and illogical data, and subsequently copied into SPSS version 13.0 software (SPSS, Chicago, IL, USA). Statistical analysis was performed by an independent statistician blinded to the patients’ surgical outcomes. One-way ANOVA test and the chi squared test were used for statistical analysis. Scheffe’s *post hoc* test was performed for comparison among groups. The independent paired sample *t*-test was applied for comparisons of functional results before and after surgery. The level of statistical significance was set as *P* < 0.05.

## Results

A total of 137 patients (198 knees) met our inclusion criteria. The final study group included 96 women and 41 men. The mean patient age was 74 years (range: 59–86 years) at the time of surgery. The mean patient height was 152.4 cm (range: 138.0–172.0 cm), the mean weight was 64.8 kg (range: 37.5–96.0 kg), and the mean body mass index was 27.6 kg/m^2^ (range: 17.6–36.0 kg/m^2^). The mean duration of follow-up was 52.2 months (range: 24–95 months).

There were 55 knees in group A, 63 knees in group B, and 80 knees in group C. Demographically, there were no statistical differences in age at time of operation, body mass index, length of hospital stay, and preoperative mechanical axes among the three groups. The tourniquet time was longest in group A patients (Table 1).

**Table 1:** **Demographic and radiographic data from patients in the conventional and CAS groups**

**Parameters**	**Group A (** ***n*** = **55)**	**Group B (** ***n*** = **63)**	**Group C (** ***n*** = **80)**	***p*** **value**
Demographic data				
Age at time of operation (year)	75 ± 5	74 ± 6	72 ± 6	0.106
Body height (cm)	151 ± 6	151 ± 6	156 ± 9^a^	0.004*
Body weight (kg)	59 ± 8	63 ± 8	67 ± 11^a^	0.011*
Body mass index (kg/m^2^)	26 ± 4	28 ± 4	28 ± 4	0.151
Follow-up (months)	49 ± 27	55 ± 25	50 ± 31	0.629
Perioperative data				
Total blood loss (mL)	549 ± 189	612 ± 220	615 ± 311	0.610
Tourniquet time (min)	85 ± 22^a^	65 ± 15	69 ± 18	<0.001*
Hospital stay (days)	7 ± 2	7 ± 2	6 ± 1	0.311
Radiographic data, leg axis				
Valgus correction angle of the distal femur (°)	11 ± 1	10 ± 1	6 ± 1^a^	<0.001*
Coronal femoral bowing angle (°)	11 ± 3	10 ± 2	3 ± 1^a^	<0.001*
Preoperative MA (°)	164 ± 4	164 ± 4	166 ± 6	0.143
Postoperative MA (°)	179 ± 2	177 ± 4^a^	179 ± 2	<0.001*
Component alignments				
Femoral valgus angle (°)	99 ± 1^a^	96 ± 2	96 ± 1	<0.001*
Femoral flexion angle (°)	2 ± 1^a^	4 ± 3	5 ± 4	0.001*
Tibial valgus angle (°)	90 ± 1	90 ± 2	90 ± 1	0.538
Tibial flexion angle (°)	88 ± 2	88 ± 2	87 ± 2	0.126

Group A: osteoarthritis with marked coronal femoral bowing; all underwent CAS-TKA. Group B: osteoarthritis with marked coronal femoral bowing; all underwent conventional TKA. Group C: osteoarthritis without marked coronal femoral bowing; all underwent conventional TKA. Values are shown as mean ± SD; *p* values for between-group comparison were determined by one-way ANOVA tests.

*Abbreviations*: *MA* mechanical axis, *AA* anatomic axis.

*Statistically significant (*p* value < 0.05); ^a^statistically significant among groups.

Significant differences in the valgus correction angle of the distal femur were detected among the three groups (*P* < 0.001). Scheffe’s *post hoc* test revealed differences between individuals in group A and group C and between those in group B and group C (*P <* 0.001 and *P <* 0.001, respectively), but no differences were noted between groups A and B (*P* = 0.987). There was no difference in the femoral bowing angle between groups A and B (*P* = 0.794), according to Scheffe’s *post hoc* test.

Differences in the postoperative mechanical axis were found among the three groups. Mean postoperative mechanical axes in groups A, B, and C were 179°, 177°, and 179°, respectively. Scheffe’s *post hoc* test revealed differences between group A and group B and between group B and group C (*P* = 0.002 and *P* = 0.003, respectively). No such differences could be detected between groups A and C (*P* = 0.534) (Table 1). Similar inter-group differences were noted when comparing the outliers of knees achieving the ideal postoperative mechanical axis in the three groups (3.7%, 50.8%, 5.0%, respectively) (Table 2).

**Table 2:** **Comparison of outliers of mechanical axis, femoral valgus angle and tibial valgus angle in the three groups**

	**No. of postoperative component alignments more than 3° deviation**			
	**Group A** ***N*** = **55**	**Group B** ***N*** = **63**	**Group C** ***N*** = **80**	***p*** **value**
Mechanical axis >3°	2 (3.7%)	32 (50.8%)	4 (5.0%)	<0.001*
Component alignments >3°				
Femoral valgus angle	3 (5.5%)	33 (52.4%)	5 (6.2%)	<0.001*
Tibial valgus angle	1 (1.8%)	4 (6.3%)	2 (2.5%)	0.335

Group A: osteoarthritis with marked coronal femoral bowing; all underwent CAS-TKA. Group B: osteoarthritis with marked coronal femoral bowing; all underwent conventional TKA. Group C: osteoarthritis without marked coronal femoral bowing; all underwent conventional TKA. The values are given as the *n* (%); *p* values for between-group comparison were determined by chi squared tests.

*Statistically significant (*p* value < 0.05).

Radiographic analysis of component alignment angles in the coronal and sagittal planes revealed no differences in TV angle among the three groups (*P* = 0.538) or TF angle (*P* = 0.126). However, there were significant differences in the FV angle in groups A, B, and C (*P* < 0.001). The FF angle was also significantly different among the three groups (*P* = 0.001) (Table 1). Scheffe’s *post hoc* test revealed statistically significant differences in the femoral cuts (FV angle and FF angle) with the use of CAS-TKA. When comparing the FV angle and FF angle, no statistically significant differences could be found between patients without bowing and those with bowing after conventional TKA (Table 1).

When we compared the alignment data between groups A and B, we found that the preoperative mechanical axis and the valgus correction angle were very similar. With regard to the postoperative alignment data, a similar tibial valgus angle was seen between patients in group A and group B. The chief difference in the coronal plane lies with the femoral valgus angle, which results in significant differences in the postoperative mechanical axis.

The mean preoperative IKS scores and the patella scores were similar in the three groups, and the patellar score improved postoperatively in all three groups. Similarly, improvement of postoperative outcomes with regard to the IKS clinical knee score, the IKS pain score, and the IKS functional knee score were also noted (Table 3). The difference in scores, however, did not achieve statistical significance among three groups (*P* > 0.05).

**Table 3:** **Comparison of preoperative and postoperative knee scores in the three groups**

**Parameters**	**Group A (** ***n*** = **55)**	**Group B (** ***n*** = **63)**	**Group C (** ***n*** = **80)**	***p*** **value**
Preoperative knee scores				
Patellar score	16.8 ± 8.4	16.4 ± 9.0	17.3 ± 9.4	0.445
IKS knee score	43.8 ± 12.8	46.4 ± 11.6	49.1 ± 13.0	0.240
IKS pain score	17.1 ± 7.8	18.5 ± 6.8	20.3 ± 5.3	0.073
IKS function score	34.5 ± 13.9	38.2 ± 10.9	38.7 ± 9.9	0.320
Postoperative knee scores				
Patella score	26.7 ± 2.7	26.2 ± 3.6	26.6 ± 3.4	0.583
IKS knee score	95.8 ± 3.1	94.7 ± 3.3	96.0 ± 4.9	0.392
IKS pain score	47.4 ± 3.0	46.2 ± 3.7	48.0 ± 4.0	0.108
IKS function score	91.9 ± 9.3	91.0 ± 9.0	93.0 ± 8.9	0.643

Group A: osteoarthritis with marked coronal femoral bowing; all underwent CAS-TKA. Group B: osteoarthritis with marked coronal femoral bowing; all underwent conventional TKA. Group C: osteoarthritis without marked coronal femoral bowing; all underwent conventional TKA. Values are shown as mean ± SD; *p* values for between-group comparison were determined by One-way ANOVA tests.

*Abbreviations*: *HSS score* Hospital for Special Surgery knee score, *IKS score* International Knee Society score.

Periprosthetic femoral fractures occurred in two knees. One fracture occurred in a patient in group A, who underwent open reduction and internal fixation with dynamic condylar screws (Synthes, Basel, Switzerland). The other knee, from a patient in group C, was managed with open reduction and internal fixation using Less Invasive Stabilization System (LISS) locking plates (Synthes, West Chester, PA, USA). Both knees healed uneventfully. No complications attributable to placement of pins for the femoral and tibial reference arrays for CAS-TKA were noted in group A. Pulmonary emboli, deep vein thrombosis, and postoperative wound infection were not encountered in this series.

## Discussion

The long-term outcome of TKA depends on good positioning of components and a reconstructed mechanical axis that is within 3° of neutral in the coronal plane [1–12]. Achieving a distal femoral bone cut perpendicular to the mechanical axis of the femur depends on the valgus correction angle of the distal femur [18]. Anatomically, the angular relationship between the mechanical and anatomical axis of the femur is influenced by bowing deformity in the coronal plane [18–22].

We are aware of few population survey studies that have specifically examined the prevalence of coronal femoral bowing in ethnic Asians; this anatomic feature has been reported to be as high as 62% in Asian patients with end-stage osteoarthritis of the knee [18–20]. Coronal alignment with marked bowing deformity is neither apparent clinically nor is evident on short-film radiographs of the knee [19,20]. The question of whether a full-length weight-bearing roentgenogram of the lower extremity should be a routine part of the preoperative protocol for TKA is still being debated [18–22]; however, we recommend it before performing TKA in ethnic Asians. It can provide excellent information about coronal malalignment and extra-articular deformities of the femur, information that is necessary for establishing an ideal reconstructed mechanical axis (Figure 1).

Most surgeons utilize a fixed 5° to 6° valgus cut angle for the intramedullary femoral cutting guide, as recommended by Kharwadkar et al. [32]. However, the presence of marked coronal femoral bowing changes the angle between the mechanical and anatomical axis, and the fixed 5° to 6° valgus cut angle no longer holds true [18–20]. In this series, the mean valgus correction angle of the distal femur was 11.1° ± 1.3° in group A patients and 10.4° ± 1.2° in group B (Table 1). Under these circumstances, utilizing a fixed 5° to 6° valgus cutting guide would have resulted in inaccurate placement of femoral components and an unacceptable reconstructed mechanical axis.

**Figure 3:**
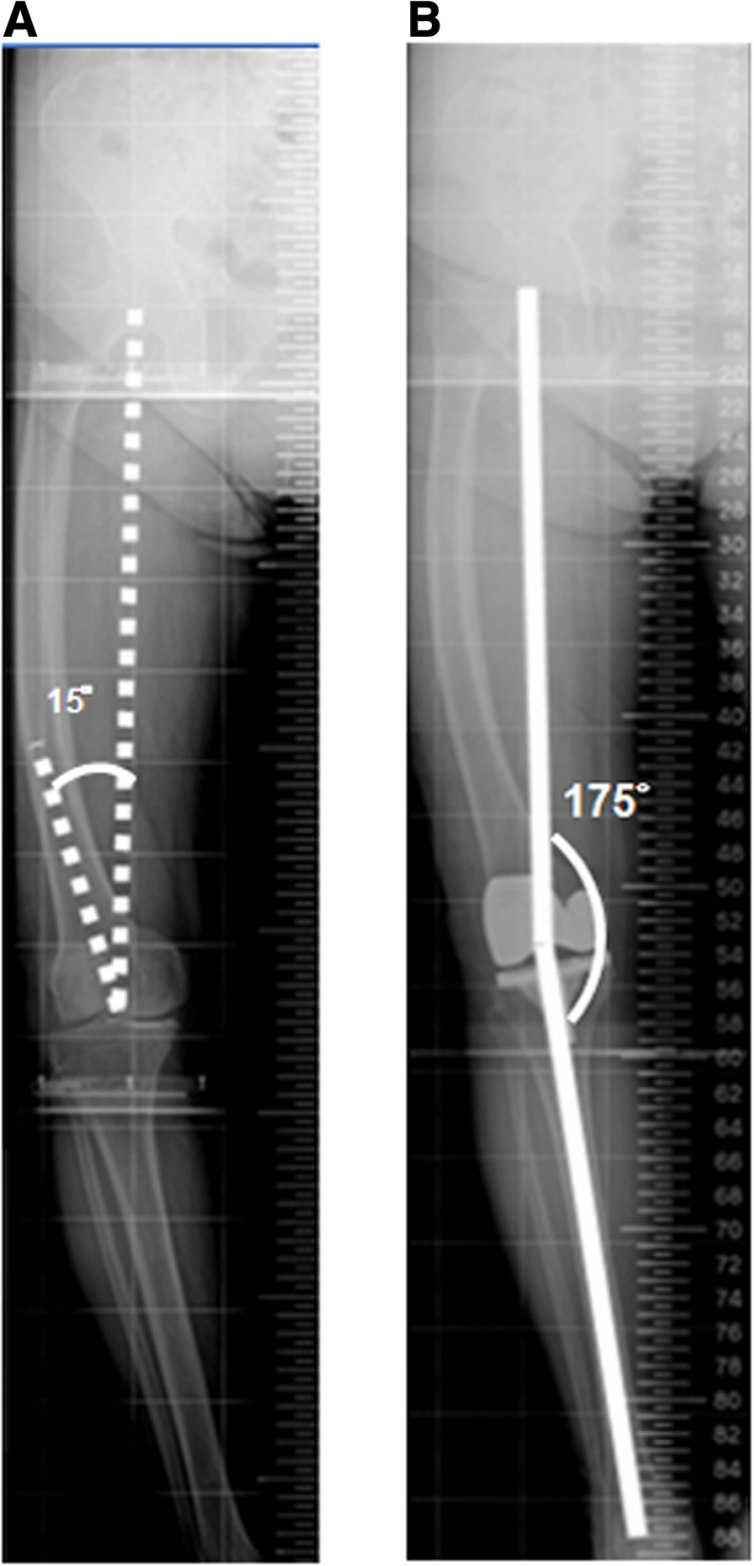
**Representative results in one patient with marked coronal femoral bowing deformity who had undergone conventional TKA. (A)** This preoperative full-length weight-bearing roentgenogram of the lower extremity shows marked coronal femoral bowing with a 15° femoral valgus resection angle. **(B)** A postoperative full-length weight-bearing roentgenogram of the lower extremity shows that the postoperative mechanical axis is 175°; this residual varus deformity was presented after conventional TKA was performed.

Any femoral cutting system and technique (intramedullary or extramedullary alignment guidance system) must incorporate appropriate preoperative planning to adjust the cuts to accommodate the deformity. However, the majority of currently available femoral jigs do not provide a broad enough choice of valgus cut angles to carry out an ideal reconstructed mechanical axis in patients with such deformities (Table 1) (Figure 3). An intra-articular bone resection technique was described by Wang and Wang [33]. Utilizing an extramedullary guide system or modification of the starting hole in knees in which an intramedullary guide system was used has been reported to be effective for arthritis of the knee with extra-articular deformity; however, it is a technically difficult approach for patients with uniplanar, biplanar, and triplanar femoral extra-articular deformity in conjunction with ipsilateral osteoarthritis of the knee [33,34]. In the current study, the intramedullary femoral guide could not provide the planned valgus correction angle. The author utilized intra-articular bone resection with modification of the starting hole of the intramedullary guidance system in the lateral femoral condyle. In group B, however, improper postoperative mechanical axis and femoral component alignment may be resulted from the subsequent erroneous distal femur resection by the incomplete insertion of intramedullary rods [35]. Patient-specific instrumentation (PSI) is a modern technique, and the theoretical benefit of PSI may be useful for end-stage arthritis of the knee joint in conjunction with extra-articular deformity [34]. However, the role of PSI in TKA has not been clearly defined in literatures [36–38].

**Figure 4:**
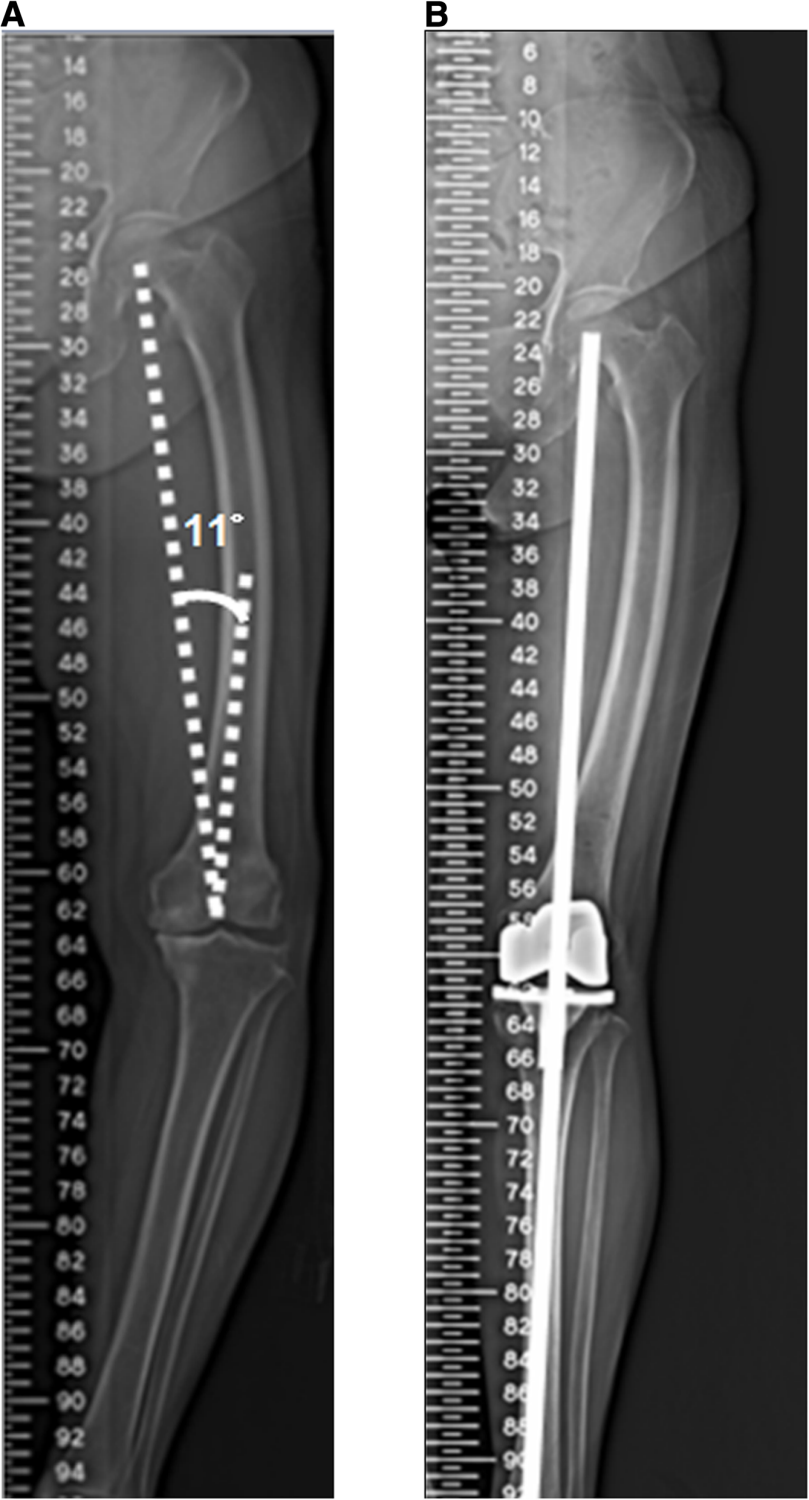
**Roentgenogram of the lower extremity showing marked coronal femoral bowing and complete restoration of limb alignment after undergoing CAS-TKA. (A)** This preoperative full-length weight-bearing roentgenogram of the lower extremity shows marked coronal femoral bowing. The distal femoral valgus resection angle was 11°. **(B)** This postoperative full-length weight-bearing roentgenogram of the lower extremity shows complete restoration of limb alignment after the patient underwent CAS-TKA.

CAS-TKA has been reported to provide more accurate bone cuts; more precise component placement in coronal, sagittal, and rotational alignment; better restoration of coronal limb alignment; and less gap asymmetry [39–41]. Mullaji et al. retrospectively analyzed 14 knees in conjunction with marked coronal bowing of the femur and found that CAS-TKA provided better results [20]. In our study, a comparison of patients in group B and group C, both groups that were treated with conventional TKA, we also found that improper reconstructed mechanical axes were significantly present in patients with coronal alignment with marked bowing. Our results with CAS-TKA in group A patients show that CAS performs better than conventional guidance systems in restoring the mechanical axis in the presence of coronal alignment with marked bowing, similar to that seen in group C (Table 1) (Figure 4). Therefore, CAS-TKA is a valuable adjunct for obtaining proper alignment in patients with coronal alignment with marked bowing, where accurate placement of prosthetic components and restoration of the mechanical axis may be challenging. One major technical limitation should be kept in mind: if the anticipated femoral condylar resection violates the integrity of the insertion of either the medial or the lateral collateral ligament, the intra-articular osteotomy should not be done. Instead, a corrective extra-articular osteotomy should be considered [33,34].

In this study, all three groups showed postoperative improvement in all four clinical outcome measures (IKS score for pain, the IKS clinical knee score, IKS functional knee score, and the patellar score). The differences in the scores among the three groups did not reach statistical significance. No statistical difference in short-term clinical assessments was demonstrated between the computer-assisted and conventional TKA groups. Therefore, long-term clinical comparisons of both techniques are worthwhile, and this warrants further investigation.

Several limitations of this study must be acknowledged. First, this was a retrospective study, with all the inherent weakness and biases of such study designs. Second, the small number of patients was studied. Although we determine the adequate sample size (51 knees were required per group) to detect a difference of IKS score, a randomized controlled trial involves large sample sizes and long-term follow-up is worthwhile to clarify if the well-aligned TKA translates to better clinical outcomes and longevity. Third, the current study was limited to Asian patients and thus to a non-western experience with total knee arthroplasty. Finally, this study is limited by its short-term clinical follow-up. We were unable to assess the correlations among proper alignment, long-term functional outcome, and survival of the prosthesis. In the moderate number of articles published comparing CAS-TKA versus conventional TKA, the short-term to mid-term results of both techniques have been good. We intend to follow up this cohort to prove or disprove the long-term clinical benefits of CAS-TKA and survival of the prosthesis.

The presence of coronal alignment with marked bowing results in loss of accuracy in reconstructed mechanical axis when a conventional intramedullary femoral guide is used. Instead of the traditional fixed 5° to 6° valgus correction angle of the distal femur, in Asian patients, the angle first needs to be determined and then adjusted by full-length weight-bearing roentgenograms of the lower extremity. Modifying the intramedullary femoral guide to provide a wider choice of valgus cut angle or using combined osteotomy or an intra-articular bone resection technique are all viable options.

## Conclusions

Our data suggest that CAS-TKA can be an effective alternative for accurate restoration of femoral alignment. However, with regard to clinical function, we were not able to show a statistically significant difference between the computer-assisted and conventional TKA. Long-term follow-up will be needed to determine if the improvement in radiographic results actually translates to better clinical outcomes.

## Abbreviations

CAS-TKA: computer-assisted surgery-total knee arthroplasty
FF: femoral flexion angle
FV: femoral valgus angle
IKS: International Knee Society
MA: mechanical axis
PSI: patient-specific instrumentation
ROM: range of motion
TV: tibial valgus angle
TF: tibial flexion angle
TKA: total knee arthroplasty
